# Intestinal Parasitic Infections in Children: A 10-Year Retrospective Study

**DOI:** 10.7759/cureus.75862

**Published:** 2024-12-17

**Authors:** Rita R Martins, Filipa Paixão, Inês Mendes, Sandra Schäfer, Isabel Monge, Francisca Costa, Paula Correia

**Affiliations:** 1 Pediatric Service, Child and Youth Department, Hospital Professor Doutor Fernando Fonseca, Lisbon, PRT; 2 Pathology Service, Hospital Professor Doutor Fernando Fonseca, Lisbon, PRT

**Keywords:** child, giardia lamblia, intestinal parasitic infections, portugal, public health

## Abstract

Background and objective

Intestinal parasitic infections are a major public health concern, especially in low-income regions with poor sanitation. Our hospital caters to a large migrant population, but data on these infections in Portugal is limited. This study aimed to assess the prevalence and characteristics of intestinal parasitic infections in pediatric patients from epidemiological, clinical, and microbiological perspectives.

Methods

A retrospective, descriptive study was conducted involving symptomatic or opportunistically screened children and adolescents, with positive stool examination for eggs, cysts, and parasites (O&P) or positive serologies for *Strongyloides*/*Schistosoma*, at a level II hospital in Portugal, between January 2012 and June 2022.

Results

Seventy-seven patients were included in the study, of whom 56% were migrants. The median age was six years. A total of 79 intestinal parasitic infections were diagnosed, corresponding to a positivity rate of 2.5%. Among these, 7.8% occurred in children aged under one year, a particularly vulnerable group. The most frequently identified parasite was *Giardia lamblia*, followed by *Strongyloides stercoralis*. The rate of diagnoses increased over the study period, linked to the growing migrant population. Clinically, most children and adolescents were asymptomatic. Additionally, there was a significant rate of loss to medical follow-up, which could contribute to transmission.

Conclusions

This study is unique in its focus on characterizing intestinal parasitic infections in the pediatric population, addressing a gap in existing research. It highlights the need for further research among children living in Europe, especially given the rising migrant population. Multicenter studies are crucial to better understand infection patterns and improve diagnostic and treatment protocols.

## Introduction

Intestinal parasitic infections represent a significant public health concern, affecting approximately 3.5 billion individuals annually and causing over 450 million health issues such as diarrhea, abdominal pain, malnutrition, general malaise, and impaired growth and physical development [1]. The prevalence of these infections is highest in low-income countries, largely due to poor health conditions. This is particularly evident in sub-Saharan Africa, Asia, and Latin America, where the expressiveness of the infections is more pronounced, resulting in significant morbidity and mortality [2].

The most prevalent helminth parasites responsible for intestinal infections are Nematodes (*Enterobius vermicularis, Ascaris lumbricoides, Trichuris trichiura, Ancylostomatidae, Strongyloides stercoralis*), Cestodes (*Taenia*) and Trematodes (*Schistosome*). The most prevalent helminth infection is caused by *Enterobius vermicularis*, which is more common among children [3]. The most prevalent protozoan parasites are *Giardia lambia*, followed by *Entamoeba histolytica*, *Cyclospora cayetanensis,* and *Cryptosporidium spp.* [2,4]. It is estimated that the prevalence of asymptomatic *Giardia lamblia* infections worldwide is approximately 3-7% [5]; however, some studies have reported rates of 20-30% in healthy children attending daycare [6], and up to 50% in developing countries.

Intestinal parasitic infections are typically transmitted through the ingestion of water or food contaminated with cysts, eggs, or parasites, in a fecal-oral manner [5]. In the case of *Schistosoma, Strongyloides stercoralis,* and *Ancylostoma duodenale*, the infection can occur by transcutaneous penetration of the larvae, although the latter can also be transmitted orally [6]. The clinical manifestations of these infections are diverse and depend on several factors, including the parasite's genotype, the amount of inoculum, and the host's age and immunity [5]. Most intestinal parasitic infections are well tolerated by immunocompetent children. They may present as asymptomatic or with a wide range of non-specific abdominal symptoms, including abdominal pain, diarrhea, flatulence, nausea, anorexia, or weight loss [4,5,6].

When diagnosing parasitic infections, it is essential to consider their specific characteristics. *Giardia lamblia* should be suspected in patients with chronic diarrhea, malabsorption, anorexia, poor weight gain, or anemia [4]. *Enterobius vermicularis* infections often present with nocturnal anal or vaginal itching and can be identified by visualizing the parasite in the perianal region [4,6]. During the larval migration phase of *Ascaris lumbricoides*, individuals may experience acute transient pneumonitis, which is characterized by fever and eosinophilia, as well as high intestinal obstruction if the parasite count is high. *Trichuris trichiura* may be associated with tenesmus, rectal prolapse, and anemia [4]. *Cryptosporidium* should be considered in cases of secretory diarrhea [7]. While these infections are rarely fatal, they can significantly impair intestinal absorption of nutrients, thereby hindering physical and mental development [2].

The diagnosis typically involves testing fecal samples for eggs, cysts, and parasites (O&P) or direct examination. To test for O&P in fecal samples, three stool samples should be collected on three different days and refrigerated at 4 ºC. The sensitivity of O&P is highest if there is a 48-hour interval between each collection since parasite excretion is intermittent [4]. If there is a strong suspicion of infection and the O&P test is negative, more sensitive tests should be conducted. These may include antigen detection assays (such as ELISA or DFA) or stool PCR, which can diagnose *Giardia, Cryptosporidium, Strongyloides stercoralis, *or* Entamoeba histolytica* [7]. In the presence of diarrheal feces, it is important to collect the liquid phase for direct observation, as it contains the greatest amount of trophozoites, ensuring valuable information is not lost [4].

In patients infected with *Schistosoma *or *Strongyloides stercoralis*, serologic testing is often useful as the stool parasite burden is often low, especially among those without intestinal symptoms. This is particularly important in the case of patients infected with *Strongyloides stercoralis,* who may only experience dermatologic and respiratory symptoms or eosinophilia [8]. The diagnosis of *Enterobius vermicularis* is unique in that the Graham technique is recommended when pinworms are not visible. This procedure involves applying tape to the perianal region in the morning before showering to collect eggs deposited by female parasites overnight. The tape is then removed and sent to the laboratory for microscopic analysis [9].

Some parasitic infections, particularly those caused by helminths that affect organs outside the intestines, may result in eosinophilia (>500 eosinophils/mm^3^) or hypereosinophilia (>1500 eosinophils/mm3). However, *Giardia lamblia *or* Enterobius vermicularis* infections do not typically cause eosinophilia, making the eosinophil count an unreliable screening tool in these cases [4]. Chronic diarrhea can lead to anemia or deficiencies in vitamin B12 or folic acid [4]. Serologic tests are often unhelpful due to their low specificity, but they are recommended for diagnosing *Strongyloides stercoralis *and Schistosomiasis [8]. Contrast abdominal radiography may reveal cylindrical images consistent with *Ascaris lumbricoides* [4]. *Cryptosporidium, Giardia, Echinococcus, *and* Trichinella* are intestinal parasites that must be reported to the Portuguese National Epidemiological Surveillance System (SINAVE).

There is limited research on the incidence of parasitic diseases in Portugal [5,10]. However, since 1990, the incidence of these infections has decreased due to improvements in hygiene, sanitation, housing, and access to healthcare [6]. A study from 1992 found a significant decline in the prevalence of helminthiases over 14 years (10.4% in 1978 vs. 1.5% in 1992). The same study also reported a greater decrease in helminthiases compared to the reduction in *Giardia lamblia* infections (16% in 1978 to 11% in 1992). This may be due to the indiscriminate use of anthelmintics in routine deworming, which became common practice in the 1970s and 1980s. Two national studies carried out between 2008 and 2011 reported a parasitism rate of 3.4% to 4%, attributed almost exclusively to *Giardia lamblia;* however, this rate may be underestimated [4]. In 2021, a study by Ribeiro et al. at our hospital, analyzing the frequency of pathologies in migrant children between 2016 and 2021, detected a rate of 7.4% of asymptomatic intestinal parasitosis in this population, with 42% of these cases corresponding to *Giardia lamblia* infections [10].

The number of foreign residents in Portugal has been steadily increasing, with 2022 recording the highest number of foreigners with residence permits since the creation of the Foreigners and Borders Service (SEF). In 2022, there were 781,915 migrants, with 30.7% of them being from Brazil and 14.2% from Portuguese-speaking African countries (in Portuguese, Países Africanos de Língua Oficial Portuguesa, or PALOP). Among the PALOP countries, Cape Verde contributed the most, accounting for 33% of the total [11]. Per the 2021 Census, Lisbon has the highest proportion of migrants (15.6%), followed by Sintra (6.1%), Cascais (4.9%), and Amadora (3.4%) [10]. The Hospital Professor Doutor Fernando Fonseca (HFF) is the referral hospital for the Sintra and Amadora regions, and Ribeiro et al.'s study found that 42.9% of migrant children treated at our hospital were from PALOP countries, while 26.9% were from Brazil. Due to the high prevalence of migrants, a screening protocol for endemic pathology was developed at HFF. This protocol includes performing O&P on all migrants, even those who are asymptomatic [10].

This study aims to address the lack of research on intestinal parasitic infections in Portugal, particularly in light of the increasing number of residents with migrant status.

## Materials and methods

We conducted a retrospective and descriptive study aimed at characterizing intestinal parasitic infections in the pediatric population living in the service area of our hospital. The study included children and young individuals (aged between zero and 18 years) who had a positive stool sample for O&P or positive serologies for *Strongyloides* and/or *Schistosoma*, at HFF (a level II hospital in the Lisbon metropolitan area), between January 2012 and June 2022. Data were collected through consultation and analysis of electronic medical records.

These diagnostic tests were performed in both symptomatic children and asymptomatic individuals as part of migrant screening. The migrant screening involves a clinical protocol developed by the pediatric service of our hospital, aimed at detecting and treating early the most prevalent pathologies in migrant children and adolescents. We excluded individuals with negative stool samples for O&P or negative serologies for *Strongyloides* and/or *Schistosoma *from the study. Additionally, individuals older than 18 years at the time of the diagnosis of intestinal parasitic infection were also excluded.

We analyzed laboratory variables (including stool sample results for O&P, serologies for *Strongyloides* and/or *Schistosoma*, and complete blood count results), as well as sociodemographic factors (such as sex, country of birth, parents’ country of birth, age at diagnosis, length of stay in Portugal at the time of diagnosis, and recent travel history), and clinical variables.

Statistical analysis was performed using Microsoft Excel® (Microsoft Corporation, Redmond, WA) to calculate median, minimum, and maximum values. The study was approved by the Hospital’s Ethics Committee. No artificial intelligence (AI) tool was used in the writing or preparation of this manuscript.

## Results

The study population included 77 children and young individuals, of whom 43 (55.8%) were male. The median age at diagnosis was six years (range: 1-17 years). Two children were diagnosed when they were under one year old (2.6%), 22 children between one and four years (28.6%), 46 children between five and 14 years (59.7%), and seven young people aged 15 years or older (9.1%). Of the children and adolescents, 44 (57.1%) were migrants: from Brazil, Portuguese-speaking African countries, or Southwest Asian countries (Table 1). The median duration of stay in Portugal at the time of diagnosis was 1.5 years (range: one month to six years). Among the 33 (42.8%) children/young individuals with Portuguese nationality, 12 (36.3%) had migrant parents, with Cape Verde (n=5, 41.7%) being the most frequent country of origin, followed by Guinea-Bissau (n=4, 33.3%). Nine (12%) patients had traveled to a low socioeconomic country in the six months before diagnosis.

**Table 1:** Sociodemographic characteristics

Characteristic		Absolute number (N=77)	Relative frequency
Gender	Male	43	55.8%
	Female	34	44.2%
Country of origin	Portugal	33	42.8%
	Guinea-Bissau	15	19.5%
	Cape Verde	8	10.4%
	Angola	7	9.1%
	São Tomé and Príncipe	6	7.8%
	Brazil	3	3.9%
	Guinea-Conakry	3	3.9%
	India	1	1.3%
	Pakistan	1	1.3%
Median age, years (range)		6 (1-17)	
Age group	Infants (up to 1 year)	2	2.6%
	Young children (1-4 years)	22	28.6%
	School-age children (5-14 years)	46	59.7%
	Adolescents (15-17 years)	7	9.1%

Between January 2012 and June 2022, a total of 2706 O&P and 380 parasitic serologies were conducted in pediatric patients at our hospital, leading to the diagnosis of 79 intestinal parasitic infections. This corresponds to a positivity rate of 2.5%. Out of the total parasitic infections, 54 were diagnosed through O&P and 25 through serological tests. There was a median of seven cases per year in the study period; range: 13 cases per year (16.9%) in 2017 and 2021 and only two in 2012 (2.6%).

**Figure 1:**
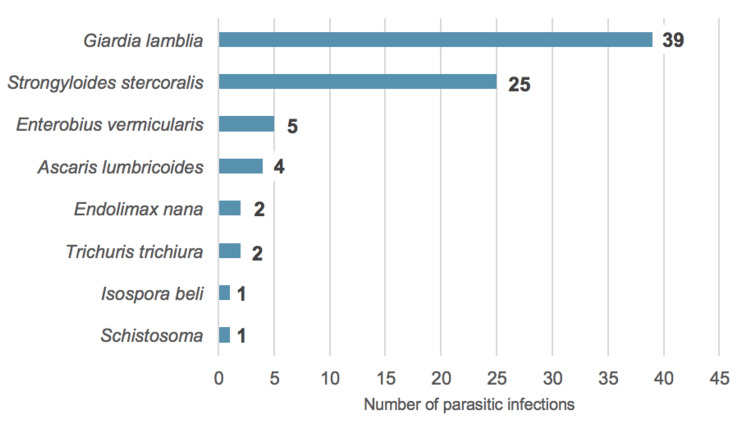
Parasites identified in the study

As shown in Figure 1, the most frequently identified parasite was *Giardia lamblia* (n=39, 50.6%), followed by *Strongyloides stercoralis* (n=25, 32.5%), *Enterobius vermicularis* (n=5, 6.5%), *Ascaris lumbricoides* (n=4, 5.2%), *Endolimax nana* and *Trichuris trichiura* (n=2, 2.6% each), and lastly, *Isospora belli* and *Schistosoma* (n=1, 1.3% each). Two patients had simultaneous infection with two parasites (one patient with *Strongyloides stercoralis* and *Isospora belli*; and another with *Ascaris lumbricoides* and *Trichuris trichiura*).

Regarding clinical presentation, the majority of patients were asymptomatic (n=44, 57.1%). Abdominal pain and diarrhea were the most frequent symptoms. The clinical characterization of the symptoms is summarized in Table 2; 31 patients (40.3%) presented with anemia and 18 patients (23.4%) with eosinophilia.

**Table 2:** Clinical presentation

Clinical presentation	Absolute number (N=77)	Relative frequency
Asymptomatic	44	57.1%
Abdominal pain	18	23.4%
Diarrhea	11	14.3%
Weight loss	6	7.8%
Anal pruritis	5	6.5%
Vomiting	3	3.9%
Anorexia	3	3.9%
Rectal bleeding	2	2.6%
Urticaria	2	2.6%

Regarding the most frequently identified parasite (*Giardia lamblia), *children aged between 5 and 14 years were the most commonly affected (n=23, 59.0%). In terms of symptoms, 18 (46.2%) children/young individuals were asymptomatic, 11 (28.2%) presented with abdominal pain, six (15.4%) with diarrhea, and four (10.3%) with weight loss. The majority (n=26, 66.7%) were migrants. Sixteen patients had anemia; 32 (82.1%) complied with therapy, and 11 (28.2%) were lost to follow-up. Six (15.4%) cases were notified in SINAVE. As for the second most frequent infection (by *Strongyloides stercoralis),* 24 cases were diagnosed through serology and one through O&P. Two (8.0%) patients had urticaria, 13 (52.0%) had anemia, and eight (33.3%) eosinophilia in blood analysis. Twelve patients (48%) were migrants.

Due to its distinctive clinical manifestation, we discuss a singular case of *Schistosoma* infection in a 13-year-old patient from Guinea-Bissau who had been residing in Portugal for less than a year. The patient was observed in the pediatric emergency department due to the presence of severe periumbilical abdominal pain, vomiting, and pasty stools. The patient was diagnosed with acute appendicitis and underwent an appendectomy. A pathological examination confirmed the diagnosis of acute appendicitis with transmural inflammation and appendicular peritonitis. Additionally, a granulomatous reaction with multinucleated giant cells within the wall was observed, which was associated with *Schistosoma* forms. A clinical reassessment two months later revealed that the young individual was asymptomatic, with microcytic anemia, eosinophilia (10,900/uL), and positive anti-schistosomiasis antibodies. Additionally, a negative stool parasitological examination and a normal abdominal ultrasound were noted. Treatment with praziquantel was recommended, but compliance could not be determined as the patient was lost to follow-up.

## Discussion

A positivity rate of 2.5% was identified regarding the diagnosis of intestinal parasitic infections during the study period. The majority of diagnoses were conducted through O&P. The positivity rate observed in our study is relatively low in comparison to other studies. In the study by Štrkolcová et al., a positivity rate of 12.7% was reported in the O&P of children aged 13-21 months residing in Slovakia [12]. Ribeiro et al. reported a prevalence rate of 7.4% of asymptomatic intestinal parasitic infections in migrant children who underwent our hospital's screening protocol for endemic pathology [10]. In a systematic review by Kantzanou et al., the overall prevalence rate of intestinal parasitic infections among children residing in European countries was 5.9%; however, significant heterogeneity was observed [13]. The prevalence rate is considerably higher in Asian and African countries, with reported rates ranging from 31.7% to 50.5%, depending on the geographical region and diagnostic methods employed [13].

Intestinal parasitic infections were more prevalent in school-age children and adolescents (68.8%), with a median age of six years. This data is in line with previous studies demonstrating a higher prevalence among school-age children (52%), compared to preschool-age (30%) [13]. In our study, 31.2% of intestinal parasitic infections were diagnosed in children aged up to four years. This is of particular relevance, given that children under the age of five are potentially more susceptible to infection due to the immaturity of their immune system [1]. The majority (57.1%) of intestinal parasitic infections were diagnosed in migrant children, mainly from African countries. Of the children born in Portugal, 36.3% had migrant parents. This finding highlights the heightened prevalence of intestinal parasitic infections in countries with low socioeconomic income [4] and in overcrowding families with low socioeconomic status [1]. However, the higher prevalence of intestinal parasitic infections in migrant children of our hospital may be attributed to the migrant screening protocol, which includes O&P.

Regarding the number of diagnoses per year, there was an increase from 2017 onwards, except in 2019 and 2020. The reduction in 2020 corresponded to the coronavirus disease 2019 (COVID-19) pandemic period and the restrictions imposed on access to scheduled healthcare. However, we found no explanation for the reduction seen in 2019. The increase in general can be attributed to the growing number of migrants residing in Portugal, particularly those utilizing our hospital services, hailing from countries with low socioeconomic status (42.9% from the PALOP) [10]. Additionally, the improvement and more consistent application of the migrant screening protocol in the pediatric department, encompassing the screening of asymptomatic patients, has contributed to this rise.

The present study identified eight species of intestinal parasites. The most frequently identified parasite was *Giardia lamblia*, representing 50.6% of cases, which aligns with another Portuguese study [6]. Although *Enterobius vermicularis* has been identified as one of the most common parasites in Portugal over the past three decades [6], we only identified five cases of it, which may be related to the collection procedure. As for *Ascaris lumbricoides,* we only identified four cases; we found a 32.5% prevalence for *Strongyloides stercoralis*, much higher than that described in a Portuguese previous study [6]. Few European studies have provided data on the prevalence of infections by *Strongyloides stercoralis*.

In a study carried out in the north metropolitan area of Barcelona, an area with dense immigration, the authors found an annual detection rate of 0.2 new diagnosed cases per 10000 inhabitants/year and one case per 10000 immigrants/year; these values are probably underestimated since the diagnosis was made mainly in patients referred for eosinophilia [14]. In a study from north-eastern Poland, in which 120 children/adolescents were tested for intestinal parasitic infections due to suggestive symptoms, the prevalence of *Strongyloides stercoralis* was 5.83% [15]. The same study reported a prevalence of *Ascaris lumbricoides* of 55.83%, *Giardia lamblia* of 12.5%, and *Enterobius vermicularis* of 3.33% [15]. A multicenter study in Turkey by Aksoy et al. revealed a prevalence of 10.1% for *Enterobius vermicularis* and 7.8% for *Giardia lamblia* [16].

A total of 40.3% of children with intestinal parasitic infections exhibited anemia, which is consistent with previous observations in the literature regarding the association between the two conditions [17]. It is well established that parasites can cause several adverse effects, including decreased nutritional intake, intestinal blood loss, splenic destruction of red blood cells, and autoimmune reactions, which can lead to a state of chronic inflammation and contribute to the anemia observed in patients with intestinal parasitic infections [17]. In our study, the main parasites associated with anemia were *Giardia lamblia* and *Strongyloides stercoralis*, which is consistent with the findings of Carrilho et al. These researchers observed a significant correlation between anemia and *Giardia lamblia, Trichuris trichiura, and Strongyloides stercoralis,* in 302 cases of intestinal parasitic infections in Brazil [18].

It is well-established that helminth infections represent one of the primary causes of eosinophilia worldwide [19]. In our study, 23.4% of patients exhibited eosinophilia, with the majority of these individuals infected by *Strongyloides stercoralis*. In the study by Webster et al. that evaluated the association between eosinophilia and intestinal parasitic infections in refugees, a higher rate of eosinophilia was found in those infected with *Strongyloides stercoralis*, which corroborates our results [19]. The majority of children diagnosed in our study were asymptomatic, as has been described in several other studies [20]. The high frequency of asymptomatic patients can be attributed to the systematic screening of migrant children in our hospital since 2015, which has enabled the diagnosis of carriers and individuals with a low parasite load. As previously documented in the literature, the most prevalent symptoms were non-specific abdominal discomfort [4,5,6].

In the context of *Giardia lamblia* infection, the highest prevalence of asymptomatic cases is observed in African countries (48.7%). Although the majority of patients completed therapy, there was a significant rate of loss to medical follow-up, which could contribute to the perpetuation of transmission. It is also noteworthy that the notification rate in SINAVE for this infection is relatively low (15.4%), considerably below expectations given that it is a mandatory notifiable disease in Portugal, as stipulated in Order No. 1150/2021 [21]. Regarding infections caused by *Strongyloides stercoralis*, our findings demonstrated that the majority of cases (24/25) were diagnosed by serology. Given the low sensitivity of O&P for this parasite, it is important to note that it may be underdiagnosed. Two patients exhibited urticaria as a symptom of this infection. In the systematic review by Tamarozzi et al., urticaria was identified as the symptom most frequently associated with Strongyloidiasis [22].

The case with acute appendicitis and in whom *Schistosoma* was detected highlights the importance of being alert to rare etiologies of common pathologies, particularly those carried over from countries of low socioeconomic status. Treatment of schistosomal appendicitis requires anti-schistosomal medication in combination with appendicectomy, as recommended in our clinical case [23].

Limitations

This study has a few limitations. Firstly, this was a single-center study confined to a single region, which may have led to an underrepresentation of the pediatric population in Portugal. Hence, the findings cannot be generalized to the broader Portuguese population. This highlights the need for multicenter studies for a more comprehensive characterization of intestinal parasitic infections in the pediatric population across Portugal. Second, the retrospective nature of the study and the fact that some patients’ medical records were incomplete may have limited the accuracy of the data. Additionally, the identification of parasites can be underestimated due to the challenges in collecting and packaging fecal samples, in addition to the fact that in some parasites, such as *Enterobius vermicularis*, eggs are rarely detected in this research. Lastly, in the context of the migrant screening protocol, this population was tested more frequently than the non-migrant population. This could have introduced a bias, as the higher number of tests may have led to an increased detection rate of intestinal parasitic infections among migrants. We recommend that in future studies testing be conducted randomly across the population, regardless of country of origin, to minimize potential biases.

## Conclusions

This study is of the utmost importance given the scarcity of studies on intestinal parasitic infections in pediatric age in Portugal, a country with a growing migrant population. This high migration rate will likely lead to an increase in the prevalence of these diseases, which are often underdiagnosed and overlooked. In the case of a migrant child or family, it is important to consider the parasites endemic in the country of origin, to provide the most appropriate complementary diagnostic tests to detect the parasite, thereby reducing false negatives. In this study, we focus on *Giardia lamblia* as the most isolated parasite and emphasize the importance of mandatory notification. Of note, eosinophilia can be an indicator of intestinal parasitic infection, particularly *Strongyloides stercoralis*, even in patients with negative O&P. It is imperative that multicenter studies be conducted to ascertain the current prevalence of intestinal parasitic infections in Portugal, as well as the associated sociodemographic factors. This will facilitate a significant improvement in the diagnosis and treatment of these conditions.

## References

[1] Fauziah N, Aviani JK, Agrianfanny YN, Fatimah SN (2022). Intestinal parasitic infection and nutritional status in children under five years old: a systematic review. Trop Med Infect Dis.

[2] Haque R (2007). Human intestinal parasites. J Health Popul Nutr.

[3] Ahmed M (2023). Intestinal parasitic infections in 2023. Gastroenterology Res.

[4] Fernandes S, Beorlegui M, Brito MJ, Rocha G (2012). Intestinal parasites protocol (Article in Portuguese). Acta Pediatr Port.

[5] (2024). General Directorate of Health. Standard nº 006/2017: Diagnostic and therapeutic approach to parasitic infections in pediatric age (Site in Portuguese). https://normas.dgs.min-saude.pt/wp-content/uploads/2019/09/abordagem-diagnostica-e-terapeutica-das-parasitoses-em-idade-pediatrica.pdf.

[6] Gata L, Gomes L, Salgado M (2013). Evolution of rates of intestinal parasites in children in Portugal (Article in Portuguese). Rev Saúde Infantil.

[7] Leder K, Weller PF (2025). Cryptosporidiosis: epidemiology, clinical manifestations, and diagnosis. UpToDate.

[8] Leder K, Weller PF (2025). Strongyloidiasis. UpToDate.

[9] Chancey R, Kamb M (2024). Chancey R, Kamb M - Centers for Disease Control and Prevention: enterobiasis/pinworm. https://wwwnc.cdc.gov/travel/yellowbook/2024/infections-diseases/enterobiasis-pinworm.

[10] Castello-Branco Ribeiro L, Paixão F, Costa F, Correia P (2024). Migrant pathology screening in the pediatric population: a five-year retrospective study from a level II hospital. Cureus.

[11] Lopes SM, Machado R (2023). Immigration, Borders and Asylum Report 2022 (Book in Portuguese). Relatório de Imigração, Fronteiras e Asilo 2022. Serviço de Estrangeiros e Fronteiras; May.

[12] Štrkolcová G, Fiľakovská Bobáková D, Kaduková M, Schreiberová A, Klein D, Halán M, Urbančíková I (2024). Intestinal parasitic infections in children from marginalised Roma communities: prevalence and risk factors. BMC Infect Dis.

[13] Kantzanou M, Karalexi MA, Vrioni G, Tsakris A (2021). Prevalence of intestinal parasitic infections among children in Europe over the last five years. Trop Med Infect Dis.

[14] Valerio L, Roure S, Fernández-Rivas G (2013). Strongyloides stercoralis, the hidden worm. Epidemiological and clinical characteristics of 70 cases diagnosed in the North Metropolitan Area of Barcelona, Spain, 2003-2012. Trans R Soc Trop Med Hyg.

[15] Zukiewicz M, Kaczmarski M, Topczewska M, Sidor K, Tomaszewska BM (2011). Epidemiological and clinical picture of parasitic infections in the group of children and adolescents from north-east region of Poland. Wiad Parazytol.

[16] Aksoy U, Akisü C, Bayram-Delibaş S, Ozkoç S, Sahin S, Usluca S (2007). Demographic status and prevalence of intestinal parasitic infections in schoolchildren in Izmir, Turkey. Turk J Pediatr.

[17] Mahmud MA, Spigt M, Bezabih AM, Dinant GJ, Velasco RB (2020). Associations between intestinal parasitic infections, anaemia, and diarrhoea among school aged children, and the impact of hand-washing and nail clipping. BMC Res Notes.

[18] Darlan DM, Ananda FR, Sari MI, Arrasyid NK, Sari DI (2018). Correlation between iron deficiency anemia and intestinal parasitic infection in school-age children in Medan. IOP Conf Ser Earth Environ Sci.

[19] Webster J, Stauffer W, Mitchell T (2022). Cross-sectional assessment of the association of eosinophilia with intestinal parasitic infection in U.S.-bound refugees in Thailand: prevalent, age dependent, but of limited clinical utility. Am J Trop Med Hyg.

[20] Elmonir W, Elaadli H, Amer A (2021). Prevalence of intestinal parasitic infections and their associated risk factors among preschool and school children in Egypt. PLoS One.

[21] (2024). Directorate-General for Health: Order No. 1150/2021: notifiable diseases to be reported on the support platform for SINAVE (National Epidemiological Surveillance System) or on SI-Vida (HIV/AIDS information system). https://files.diariodarepublica.pt/2s/2021/01/019000000/0013700190.pdf.

[22] Tamarozzi F, Martello E, Giorli G (2019). Morbidity associated with chronic Strongyloides stercoralis infection: a systematic review and meta-analysis. Am J Trop Med Hyg.

[23] Doudier B, Parola P, Dales JP, Linzberger N, Brouqui P, Delmont J (2004). Schistosomiasis as an unusual cause of appendicitis. Clin Microbiol Infect.

